# The Stroke Riskometer™ App: Validation of a data collection tool and stroke risk predictor

**DOI:** 10.1111/ijs.12411

**Published:** 2014-12-10

**Authors:** Priya Parmar, Rita Krishnamurthi, M Arfan Ikram, Albert Hofman, Saira S Mirza, Yury Varakin, Michael Kravchenko, Michael Piradov, Amanda G Thrift, Bo Norrving, Wenzhi Wang, Dipes Kumar Mandal, Suzanne Barker-Collo, Ramesh Sahathevan, Stephen Davis, Gustavo Saposnik, Miia Kivipelto, Shireen Sindi, Natan M Bornstein, Maurice Giroud, Yannick Béjot, Michael Brainin, Richie Poulton, K M Venkat Narayan, Manuel Correia, António Freire, Yoshihiro Kokubo, David Wiebers, George Mensah, Nasser F BinDhim, P Alan Barber, Jeyaraj Durai Pandian, Graeme J Hankey, Man Mohan Mehndiratta, Shobhana Azhagammal, Norlinah Mohd Ibrahim, Max Abbott, Elaine Rush, Patria Hume, Tasleem Hussein, Rohit Bhattacharjee, Mitali Purohit, Valery L Feigin, Stroke Riskometer™ Collaboration Writing Group

**Affiliations:** 1 AUT UniversityNZ; 2 Erasmus University, Medical CenterRotterdam, The Netherlands; 3 Research Center of Neurology, RAMSRussia; 4 Department of Medicine, School of Clinical Sciences, Monash UniversityAustralia; 5 Lund UniversitySweden; 6 Beijing Neurosurgical InstituteChina; 7 Stroke Foundation of BengalIndia; 8 University of AucklandNZ; 9 Universiti Kebangsaan Malaysia Medical CenterKL, Malaysia; 10 University of MelbourneAustralia; 11 University of TorontoCanada; 12 Karolinska InstitutetSweden; 13 Tel-Aviv UniversityIsrael; 14 Centre Hospitalo-UniversitaireDijon, France; 15 University of BurgundyFrance; 16 Danube UniversityAustria; 17 Otago UniversityNZ; 18 Emory UniversityAtlanta, USA; 19 Hospital de Santo AntónioPortugal; 20 University Hospital of CoimbraPortugal; 21 National Cerebral and Cardiovascular CenterJapan; 22 Mayo ClinicUSA; 23 NIH/NHLBIUSA; 24 University of SydneyAustralia; 25 Christian Medical CollegePunjab, India; 26 School of Medicine and Pharmacology, The University of Western AustraliaAustralia; 27 Department of Neurology, Janakpuri Super Speciality HospitalNew Delhi, India

**Keywords:** prevention, stroke prediction, Stroke Riskometer™ App, validation

## Abstract

**Background:**

The greatest potential to reduce the burden of stroke is by primary prevention of first-ever stroke, which constitutes three quarters of all stroke. In addition to population-wide prevention strategies (the ‘mass’ approach), the ‘high risk’ approach aims to identify individuals at risk of stroke and to modify their risk factors, and risk, accordingly. Current methods of assessing and modifying stroke risk are difficult to access and implement by the general population, amongst whom most future strokes will arise. To help reduce the burden of stroke on individuals and the population a new app, the Stroke Riskometer™, has been developed. We aim to explore the validity of the app for predicting the risk of stroke compared with current best methods.

**Methods:**

752 stroke outcomes from a sample of 9501 individuals across three countries (New Zealand, Russia and the Netherlands) were utilized to investigate the performance of a novel stroke risk prediction tool algorithm (Stroke Riskometer™) compared with two established stroke risk score prediction algorithms (Framingham Stroke Risk Score [FSRS] and QStroke). We calculated the receiver operating characteristics (ROC) curves and area under the ROC curve (AUROC) with 95% confidence intervals, Harrels C-statistic and D-statistics for measure of discrimination, R^2^ statistics to indicate level of variability accounted for by each prediction algorithm, the Hosmer-Lemeshow statistic for calibration, and the sensitivity and specificity of each algorithm.

**Results:**

The Stroke Riskometer™ performed well against the FSRS five-year AUROC for both males (FSRS = 75·0% (95% CI 72·3%–77·6%), Stroke Riskometer™ = 74·0(95% CI 71·3%–76·7%) and females [FSRS = 70·3% (95% CI 67·9%–72·8%, Stroke Riskometer™ = 71·5% (95% CI 69·0%–73·9%)], and better than QStroke [males – 59·7% (95% CI 57·3%–62·0%) and comparable to females = 71·1% (95% CI 69·0%–73·1%)]. Discriminative ability of all algorithms was low (C-statistic ranging from 0·51–0·56, D-statistic ranging from 0·01–0·12). Hosmer-Lemeshow illustrated that all of the predicted risk scores were not well calibrated with the observed event data (*P* < 0·006).

**Conclusions:**

The Stroke Riskometer™ is comparable in performance for stroke prediction with FSRS and QStroke. All three algorithms performed equally poorly in predicting stroke events. The Stroke Riskometer™ will be continually developed and validated to address the need to improve the current stroke risk scoring systems to more accurately predict stroke, particularly by identifying robust ethnic/race ethnicity group and country specific risk factors.

## Introduction

Despite a steady decrease in stroke mortality over the last two decades 1, the global burden of stroke is increasing. Almost 17 million people are affected by stroke every year (68% increase from 1990) and there were 33 million stroke survivors in the world in 2010 (84% increase from 1990), many with disability 2. Unlike 30–40 years ago when most strokes occurred in people aged ≥75 years, now most (>60%) strokes affect people younger than 75 years 2. This, together with the global epidemic of major stroke risk factors 3,4, including diabetes 5 and overweight 6, suggests that the burden of stroke is likely to increase in the future, unless more effective prevention strategies are implemented.

As most (>70%) strokes are first-ever strokes, the prevention of first-ever stroke is a major priority. The two main approaches to the prevention of first-ever stroke are the population-wide ‘mass’ approach (reducing the level of exposure to stroke risk factors in all people in the region regardless of the individual's level of risk factors), and the individual-based ‘high risk’ approach. The ‘high-risk’ aims to identify individuals at risk of stroke (e.g. people with elevated blood pressure, dyslipidaemia, atrial fibrillation and carotid artery stenosis), and to modify their risk factors, and risk, accordingly [current methods of assessing stroke risk include two established stroke risk score prediction algorithms – the Framingham Stroke Risk Score (FSRS) 7 and QStroke 8 ]. Although those with high-risk stroke benefit most from prevention strategies, the highest number of strokes and cardiovascular disease occur in people with only a mildly increased risk 9–11, mainly because there are greater numbers of people in this category of risk [according to Dalton *et al*. 12, about 90% of UK people aged 40–74 have low 10-year risk of stroke (<20%) as determined by QRisk2]. However, the general population, amongst whom most future strokes will arise, do not readily access and utilize these prediction models; the vast majority of people do not know their risk of having a stroke, do not know their risk factors, and do not know what to do about it 13–15.

Recent advances in mobile (smartphone) technologies and their worldwide use (about 1·4 billion users) offer unique opportunities to utilize these technologies for improving health and reducing burden from these disorders. Importantly, easily accessible and cost-effective risk-estimation systems are well suited to the developing world and other regions where access to medical facilities is limited 16, including elderly populations where smartphones are being increasingly used 17–19.

In recognition of the importance of e-research into noncommunicable disease (NCD) initiatives, the United Nations (UN) Economic and Social Council, the International Telecommunication Union (ITU) and the World Health Organization (WHO) have recently (June 2013) launched a new mHealth initiative for improving NCD prevention, treatment and policy enforcement 20. In order to inform and support these UN/ITU/WHO efforts, and to increase general awareness about stroke and its risk factors as well as to improve stroke and NCD prevention on an individual level, The National Institute for Stroke and Applied Neurosciences, AUT University recently developed an app called the Stroke Riskometer™. This app utilizes recent advances in risk presentation/communication 21,22, international guidelines on stroke and CVD prevention 23–28 and has the potential to significantly improve stroke and NCD prevention 29. The Stroke Riskometer™ algorithm was derived from the Framingham Stroke Risk Score (FSRS) prediction algorithm 7 and enhanced to improve accessibility and to include several additional major risk factors shown to be important for stroke, largely based on the INTERSTROKE study 4.

Endorsed by the World Stroke Organization, World Federation of Neurology and International Association on Neurology and Epidemiology, the app provides estimates of the absolute risk of stroke within the next 5 and 10 years for individuals aged ≥20 years. Importantly, the Stroke Riskometer™ provides not only their absolute risk of stroke development but also a baseline risk for comparison, thus allowing users to compare their risk of stroke with someone of the same age and gender who has no risk factors. The former represents a new paradigm for high-risk stroke prevention strategy 29, and enables a refined presentation of the traditional threshold-based approach in which people are categorized into low, moderate, and high-risk groups. This procedure enables not only those at high levels of risk, but also those at low- to moderate absolute risk, to reduce their risk of stroke. The app therefore allows a combination of both high-risk and population strategies, an approach shown to be the most effective for cardiovascular disease prevention 11.

The aim of this study was to compare the performance of the Stroke Riskometer™ prediction algorithm with two other commonly used stroke prediction algorithms – Framingham Heart Study Stroke Risk Score (FSRS) prediction algorithm 7 and QStroke 8.

## Methods

### Study design and data sources

Three study populations (80 308 person-years of observation in total) were used to validate the Stroke Riskometer™ algorithm: the Auckland Regional Community Stroke (ARCOS IV) 2011–2012 study 30, the Rotterdam Study (1990 – ongoing) 3,31, and Russian Cohort studies (1992 – ongoing; Dr M Kravchenko, unpublished data).

The ARCOS study is a population-based stroke register where all new stroke events (both hospitalized and nonhospitalized, fatal and nonfatal) in almost 1·2 million Auckland adult residents were prospectively ascertained using multiple overlapping sources of the information, including hospital admissions/referrals, community general practices and death certificates etc. (details of the study methodology have been described elsewhere) 30. For the purpose of the validation of the Stroke Riskometer™ we used a sub-set of ARCOS IV data on strokes in people aged 21–95 years (*n* = 410).

The Rotterdam Study has been described previously 3. It is an ongoing prospective population-based cohort study that focuses on the causes and consequences of chronic and disabling diseases in the elderly 31. The cohort started enrolment in 1990 and included 7983 participants aged ≥55 years living in Ommoord, a district of the city of Rotterdam in the Netherlands (participation rate 78%). Follow-up was complete until January 1, 2012, for 97·1% of potential person-years 32. The Rotterdam study contributed data from *n* = 7713 individuals who ranged in age from 55–90 years.

Russian cohort studies were conducted in Moscow (*n* = 412), Ulyanovsk (*n* = 512), Nal'chik (*n* = 177) and Minsk (*n* = 277) over various time periods starting from 1992. Study participants (men and women; age range 39–66 years) were followed up from 12 years (Moscow) to four-years (Ulyanovsk, Nal'chik and Minsk). The World Health Organization stroke diagnostic criteria 33 were used and a diagnosis of stroke was confirmed by a study neurologist across all these studies (over 90% of stroke patients had brain neuroimaging to establish a pathological type of stroke). All these studies have been approved by the local Ethics Committees.

### Stroke risk factors and algorithm development

Risk scores from three stroke predictors were generated. Each scoring algorithm utilized a series of known or hypothesized stroke risk factors (Table 1), some of which are in addition to those used in the FSRS and are the central targets in the new WHO Global Action Plan for the NCD 2013–2020 34. Distribution of each risk factor for each data set is listed in Table 2. The Stroke Riskometer™ algorithm was derived from the Framingham Stroke Risk Score (FSRS) prediction algorithm 7 but enhanced to include several additional major risk factors shown to be important for both ischaemic and haemorrhagic strokes, largely based on the INTERSTROKE study 4. The additional variables are listed in Table 1. Questions were based on recall such as ‘Have you ever been told by a doctor that you have atrial fibrillation (irregular heartbeats)?’ and ‘Have you ever been told by a doctor that you have left ventricular hypertension?’ such that no immediate medical test (e.g. an ECG is required) in order for users to provide an answer. These questions have been used and validated in cross-sectional studies 4. Beta-coefficients for each additional variable were derived from current literature and discussed amongst by a panel of stroke and health experts of the Stroke Riskometer™ Collaboration. Based on these discussions and available evidence, the following risk scores were added to the FSRS 7 risk score: 0·20 for being non-Caucasian 23,35, 0·20 for poor diet (i.e., consuming less than six servings of fruits and vegetables per day) 4, 0·10 for high alcohol consumption (i.e., consuming two or more standard drinks per day) 4,36,37, 0·10 for low physical activity (i.e., less than 2·5 hours per week) 15,23, 0·05 for family history of stroke or heart attack 23,38–41, 10 (for 5-year risk) and 15 (for 10-year risk) for previous stroke or transient ischaemic attack (TIA) 42, 1·80 for any cognitive problems and 1·40 for memory problems but no cognitive issues 43, 1·20 for previous traumatic brain injury 44, 0·20 plus 0·10 for any unit (0·01) increase in waist-to-hip ratio above 0·96 for males and 0·80 for females 45. In the absence of waist-to-hip ratio data we used BMI and scored 1·02 plus 0·10 for every unit (1 kg/m^2^) above 24 kg/m^2^ for Chinese, or above 23 kg/m^2^ for South Asians or above 25 kg/m^2^ for all other ethnicities 46 [different cut-off criteria for Chinese people were based on recommendations from the Chinese National Centre for Cardiovascular Disease (W. Wang, personal communication)]. In the absence of both waist-to-hip ratio and BMI data, waist circumference measures can be used adding 1·02 per unit (1 cm) above 103 cm for males and 89 cm for females 45. As each of the additional risk factors was added to the algorithm separately without taking into account interactions between the risk factors, we applied conservative beta-estimates to reduce the chance of overestimating the stroke risk 47,48. Algorithm testing prior to the app launch used a number of different methods. A very large number of hypothetical cases (many hundreds of different combination of risk factors) were entered into the tool to identify problems requiring resolution before clinical use. The tool then underwent clinical evaluation by stroke experts and general practitioners to compare the estimated 5-year and 10-year risk.

**Table 1:** Stroke Riskometer™ variables

Variables	Definition
Age*	In years
Gender*	Males or Females
SBP*	Systolic blood pressure measured in mm/Hg
Antihypertensive treatment*	Any blood pressure lowering medications or antihypertensive medicines No = 0, Yes = 1
Diabetes*	Yes = 1, No = 0
CVD risk*	History of CVD (heart attack or peripheral artery disease) Yes = 1, No = 0
Smoking status*	Never, Ex-Smoker, Current
Atrial fibriliation*	Yes = 1, No = 0
Left ventricular hypertrophy by ecg*	Yes = 1, No = 0
Family history of stroke or heart attack*	Yes = 1, No = 0
**Alcohol consumption**	More than 2 standard drinks per day.
**Stress**	Significant stress as determined by the patient. Diagnosis of anxiety or depression.
**Low physical activity**	Less than 2·5 hours per week.
**Waist to hip ratio (WHR)**	In males, if WHR > 0·96 then add 0·20 + 0·10 for every unit (0·01) above this threshold In females, if WHR > 0·80 then add 0·20 + 0·10 for every unit (0·01) above this threshold
**Non-Caucasian**	Caucasian = 0, Non-Caucasian = 1
**Poor diet**	Less than six servings of fruit and vegetable per day = 1, More than or equal to six servings of fruit and vegetables per day = 0
**Cognitive problems or dementia**	Yes = 1, No = 0
**Poor memory**	No cognitive problems but has poor memory Yes = 1, No = 0
**Previous TBI**	Previous Traumatic Brain Injury Yes = 1, No = 0
**BMI**	If WHR not available. We added 0·10 for every unit (1) above 24 kg/m^2^ for Chinese, or above 23 kg/m^2^ for South Asians or above 25 kg/m^2^ for all other ethnicities
**Waist circumference**	If WHR and BMI not available. We added 1·02 per unit (1 cm) above 103 cm waist circumference for males and 89 cm for females

Variables denoted with an asterix (*) comprise the existing Framingham Stroke Risk Score (FSRS) algorithm where the beta-coefficients differ for males and females. Variables in bold are new additions to the Stroke Riskometer™.

**Table 2:** Baseline characteristics of the validation cohorts (ARCOS, RUSSIA and ROTTERDAM) and which variables are required for each of the three risk score algorithms being assessed

Algorithm	Variables		Data set					
			ARCOS (*n* = 410)		RUSSIA (*n* = 1378)		ROTTERDAM (*n* = 7713)	
			Males	Females	Males	Females	Males	Females
F, R, Q	Age (years)		68·8 (13·2)	72·4 (15·7)	50·3 (6·2)	50·6 (6·4)	69·0 (8·7)	71·7 (10·2)
F, R, Q	SBP (mmHg)	Mean (SD)	156·8 (30·1)	157·3 (29·9)	135·8 (19·4)	130·8 (21·1)	138·7 (21·8)	140·0 (22·8)
R	Waist-to-hip ratio		0·9 (0·1)	0·9 (0·1)				
F, R, Q	BMI (kg/m^2^)				27·8 (4·3)	27·5 (5·4)	25·6 (2·9)	26·7 (3·7)
R	Waist circumference (cm)		97·2 (15·9)	99·3 (14·5)				

		Categories	N (%)	N (%)	N (%)	N (%)	N (%)	N (%)
	Stroke Event		216 (100%)	194 (100%)	21 (4·4%)	24 (2·7%)	268 (8·6%)	407 (8·3%)
F, R, Q	Anti-hypertensive medication		172 (79·6%)	154 (79·4%)	162 (33·8%)	321 (35·8%)	915 (29·5%)	1728 (35·5%)
F, R	Diabetes		42 (19·8%)	39 (20·3%)	21 (4·4%)	43 (4·8%)	184 (6·1%)	331 (7·1%)
F, R, Q	Atrial Fibrillation		59 (27·3%)	87 (44·9%)	47 (9·8%)	69 (7·7%)	170 (5·5%)	208 (5·1%)
F, R, Q	Left ventricular hypertrophy		0 (0%)	0 (0%)	101 (26·8%)	130 (19·2%)	144 (4·8%)	194 (4·9%)
F, R, Q	History of CVD		100 (46·5%)	93 (47·9%)	57 (11·9%)	95 (10·6%)	752 (24·2%)	903 (18·5%)
R	Previous stroke or TIA		66 (30·6%)	62 (32·0%)	33 (6·9%)	33 (3·7%)	407 (13·1%)	704 (14·4%)
Q	Rheumatoid arthritis						26 (2·4%)	92 (4·7%)
Q	Chronic kidney disease						339 (16·8%)	661 (20·3%)
Q	Chronic coronary heart failure		22 (10·4%)	21 (11·0%)				
R	Cognitive problems or dementia						114 (3·9%)	368 (8·0%)
R	Poor memory						586 (19·2%)	1010 (21·6%)
R	Previous TBI						1071 (35·3%)	1270 (27·4%)
F, R, Q	Family history of stroke/heart attack		133 (61·6%)	112 (57·8%)	240 (49·9%)	492 (54·9%)	1548 (49·9%)	2523 (51·7%)
R, Q	Non-European		46 (21·3%)	46 (23·7%)	0 (0%)	0 (0%)	40 (1·4%)	56 (1·3%)
R, Q	Race-ethnicity	European	170 (78·7%)	148 (76·3%)	481 (100%)	897 (100%)	2846 (98·6%)	4281 (98·7%)
		Maori	5 (2·3%)	10 (5·2%)	0 (0%)	0 (0%)	0 (0%)	0 (0%)
		Pacific	9 (4·2%)	20 (10·3%)	0 (0%)	0 (0%)	0 (0%)	0 (0%)
		Chinese	7 (3·2%)	4 (2·1%)	0 (0%)	0 (0%)	0 (0%)	0 (0%)
		South Asian	8 (3·7%)	3 (1·6%)	0 (0%)	0 (0%)	0 (0%)	0 (0%)
		Other	17 (7·9%)	9 (4·6%)	0 (0%)	0 (0%)	40 (1·4%)	56 (1·3%)
R	Poor diet		102 (47·2%)	104 (53·6%)			1362 (62·2%)	1808 (56·7%)
R	Low physical activity		176 (81·5%)	154 (79·4%)	224 (46·7%)	430 (47·9%)		
R	High alcohol consumption		118 (54·6%)	104 (53·6%)	106 (22·0%)	14 (1·6%)		
R	Experienced stress		52 (24·1%)	39 (20·1%)	141 (29·5%)	470 (52·8%)		
F, R	Smoking	Yes	114 (52·8%)	88 (45·4%)	202 (42·0%)	137 (15·3%)	920 (29·63%)	805 (16·50%)
Q	Smoking status	Ex-Smoker/Light	77 (35·8%)	69 (36·1%)	117 (24·3%)	82 (9·1%)	2058 (66·28%)	1503 (30·81%)
		Current-Smoker/Moderate	34 (15·8%)	17 (8·9%)	18 (3·7%)	9 (1·0%)	294 (9·47%)	318 (6·52%)
		/Heavy			202 (42·0%)	137 (15·3%)	270 (8·69%)	216 (4·43%)

Important new variables required for the Stroke Riskometer™ algorithm but were not present in the data set assessed are represented by shaded grey boxes.

F, Framingham Stroke Risk Score (FSRS), R, Stroke Riskometer™, Q, Qstroke.

### Algorithm validation

The performance of the Stroke Riskometer™ was tested across three data sets (ARCOS, Russian and Rotterdam) as greater precision is gained when assessing risk prediction models using multiple epidemiologic studies compared to single-studies 49. We also compared performance of the Stroke Riskometer™ with the FSRS 7 and QStroke 8 risk score equations. The five-year estimated risk of stroke for Russian and Rotterdam cohorts was calculated across the three different prediction algorithms. Estimates for 10-year stroke risk score were generated only for the Rotterdam study where data were available over a span of 10 years. Follow-up data for the ARCOS were limited to one-year and for Russian data sets – 4 to 12 years. We calculated Harrels C-statistic and Somer's D-statistic to measure discrimination (the ability of the algorithms to discriminate between stroke and nonstroke events). C-statistic values of 0·50 represent chance and 1 denotes the ability of the risk score to discriminate perfectly. D-statistics over 0·10 indicate that the risk score has a good ability to differentiate between an event and nonevent. Receiver operating characteristics (ROC) curve, Area Under the ROC Curve (AUROC) with 95% confidence intervals within each data set, sensitivity and specificity of each algorithm were also analyzed. R^2^ statistic was calculated to indicate the level of variability accounted for by each prediction algorithm. Calibration was assessed using the H-L test (for goodness-of-fit statistics to examine differences between the observed and predicted risks from each algorithm) All analyses were performed in R (version 3·0·2) 50.

## Results

### Validation cohorts

A total of 752 new strokes that developed in a sample of 9501 individuals over the follow-up period (80 308 person-years of observation) across three studies (ARCOS, Russia and Rotterdam) were utilized to investigate the recently derived stroke risk prediction tool algorithm Stroke Riskometer™ against two established stroke risk score prediction algorithms [FSRS 7 and QStroke 8 ]. The three data sets differed in their distribution of stroke outcomes and predictor variables required for each algorithm. The ARCOS data set was comprised of stroke only data whilst the Russian database was generated through a new cohort study, with 3·2% total strokes being observed. Of the Rotterdam study, 8·4% was comprised of strokes.

None of the three studies had all variables required for the Stroke Riskometer™ algorithm. The Russian data set was the most recent of the three data sets analyzed here so the average age was lowest (50 years for males and females; Table 2). Individuals in ARCOS and the Rotterdam study were similar in age (males 69 years and females 72 years). The Russian data set had the lowest average systolic blood pressure (SBP) while ARCOS had the highest. Both the Rotterdam and ARCOS studies had similar SBP values for males and females whilst the Russian data set had higher values in males. BMI was not recorded in ARCOS but was similar in males and females of the Russian data set (average for males = 27·8 kg/m^2^ and females = 27·5 kg/m^2^) and comparable with males and females in the Rotterdam study (average for males = 25·6 kg/m^2^ and females = 26·7 kg/m^2^). As BMI was not recorded in ARCOS waist circumference was used (for the Stroke Riskometer™ algorithm); women had greater waist circumference (99 cm) than men (97 cm). Due to inclusion of only patients with stroke, the ARCOS database had the highest percentage of individuals with diabetes (20%) compared to the Russian data set (range 4·4–4·8%) and Rotterdam (range 6·1–7·1%). A much higher proportion of the ARCOS database had individuals with a history of CVD, previous stroke/TIA event and were of non-European descent, compared to the Russian and Rotterdam cohorts (Table 2).

### Validation and overall performance of the Stroke Riskometer™

**Figure 1:**
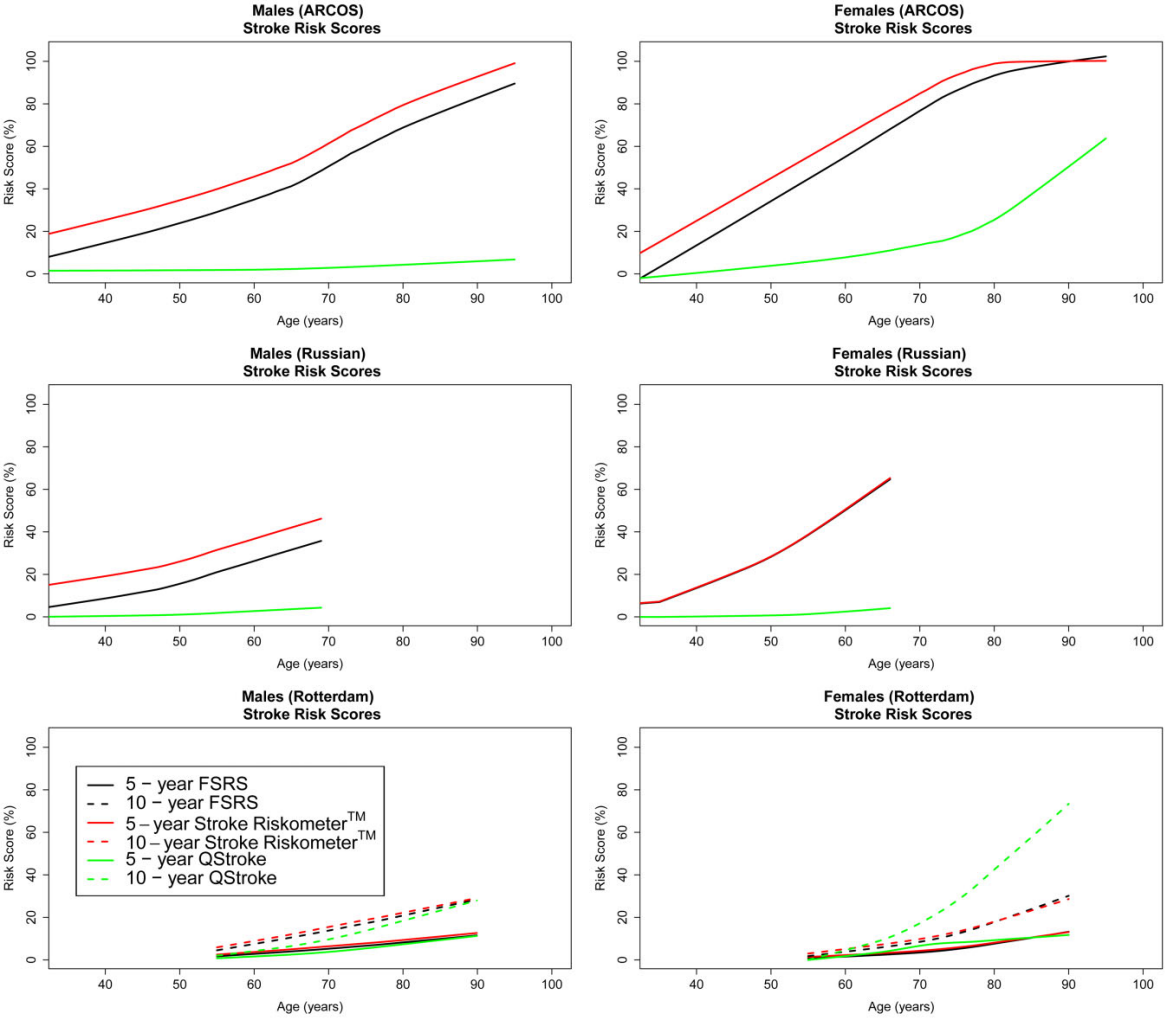
Mean predicted risk score by age for Framingham Stroke Risk Score (FSRS) (black), Stroke Riskometer™ (red) and QStroke (green) for five-years for males and females.

As none of the three studies had all variables required for the Stroke Riskometer™ algorithm, we cannot fully validate this algorithm with the emphasis for the continuing development of the Stroke Riskometer™ algorithm. We present measures of overall performance, discrimination and calibration of the Stroke Riskometer™ algorithm based on available data. The FSRS and Stroke Riskometer™ algorithms gave comparable 5-year and 10-year risk scores for males and females within each data set (Fig. 1). Risk scores differed substantially by data set, reflecting the availability of predictors within each cohort. Each algorithm (FSRS, Stroke Riskometer™ and QStroke) explained 50% of the variation observed in the ARCOS data set (R^2^ statistic, Table 3). With fewer stroke outcomes in the Russian and Rotterdam data sets, the reported R^2^ was low across all cohorts for all algorithms, ranging from 0·31–5·22% (Table 3).

**Table 3:** Validation statistics for Framingham Stroke Risk Score (FSRS), Stroke Riskometer™ and the Qstroke algorithm across all validation cohorts (ARCOS, RUSSIA and ROTTERDAM). Harrels C-statistic and Somer's D-statistic to measure discrimination (the ability of the algorithms to discriminate between stroke and nonstroke events). C-statistic values of 0·50 represent chance and 1 denotes the ability of the risk score to discriminate perfectly. D-statistics over 0·10 indicate that the risk score has a good ability to differentiate between an event and nonevent. AUROC = Area Under the Receiver operating characteristics Curve (AUROC) with 95% confidence intervals. R^2^ statistic was calculated to indicate the level of variability accounted for by each prediction algorithm

Algorithm	Year	Statistic	ARCOS		RUSSIA		ROTTERDAM	
			Mean (95% CI)		Mean (95% CI)		Mean (95% CI)	
			Males	Females	Males	Females	Males	Females
FSRS	5	R^2^ (%)	49·85 (49·73–50·08)	49·90 (49·88–49·92)	0·99 (0·08–3·14)	0·32 (0·001–1·54)	0·72 (0·21–1·81)	1·85 (0·89–3·40)
		C statistic	Stroke event data only		0·515 (0·514–0·516)	0·506 (0·505–0·506)	0·511 (0·511–0·511)	0·511 (0·511–0·512)
		D statistic			0·030 (0·029–0·031)	0·011 (0·011–0·011)	0·022 (0·021–0·022)	0·023 (0·023–0·023)
		AUROC			68·1 (58·5–77·7)	60·1 (49·1–72·0)	63·0 (57·9–68·0)	64·7 (60·1–69·4)
	10	R^2^ (%)					0·91 (0·34–1·85)	2·05 (1·17–3·23)
		C statistic					0·518 (0·517–0·518)	0·521 (0·521–0·521)
		D statistic					0·035 (0·035–0·035)	0·042 (0·042–0·043)
		AUROC					61·2 (57·6–64·8)	64·2 (61·0–67·3)
Stroke Riskometer™	5	R^2^ (%)	49·85 (49·73–50·08)	49·90 (49·88–49·92)	0·99 (0·08–3·14)	0·32 (0·001–1·54)	0·72 (0·21–1·81)	1·85 (0·89–3·40)
		C statistic	Stroke event data only		0·515 (0·514–0·516)	0·514 (0·513–0·514)	0·511 (0·511–0·511)	0·513 (0·512–0·513)
		D statistic			0·030 (0·029–0·031)	0·029 (0·028–0·029)	0·022 (0·022–0·023)	0·027 (0·026–0·027)
		AUROC			68·1 (58·5–77·7)	77·4 (69·2–85·6)	63·6 (58·5–68·5)	65·4 (61·0–69·7)
	10	R^2^ (%)					0·91 (0·34–1·85)	0·91 (0·34–1·85)
		C statistic					0·517 (0·517–0·517)	0·522 (0·521–0·522)
		D statistic					0·033 (0·032–0·033)	0·045 (0·044–0·045)
		AUROC					60·4 (58·8–64·0)	64·6 (61·6–67·6)
QStroke	5	R^2^ (%)	49·79 (49·73–50·3)	49·98 (49·88–50·04)	5·22 (1·54–14·22)	2·49 (0·24–9·77)	1·04 (0·43–2·13)	1·26 (0·60–2·36)
		C statistic	Stroke event data only		0·526 (0·524–0·527)	0·511 (0·511–0·512)	0·513 (0·513–0·513)	0·515 (0·515–0·515)
		D statistic			0·051 (0·050–0·052)	0·023 (0·022–0·023)	0·027 (0·027–0·027)	0·031 (0·030–0·031)
		AUROC			80·6 (72·3–88·9)	71·2 (59·2–83·8)	66·1 (61·9–70·2)	69·7 (66·3–73·1)
	10	R^2^ (%)					0·97 (0·41–1·92)	0·97 (0·41–1·92)
		C statistic					0·520 (0·519–0·520)	0·526 (0·526–0·526)
		D statistic					0·039 (0·039–0·039)	0·053 (0·053–0·053)
		AUROC					62·5 (59·4–65·7)	67·6 (65·0–70·1)

### Discrimination

**Figure 2:**
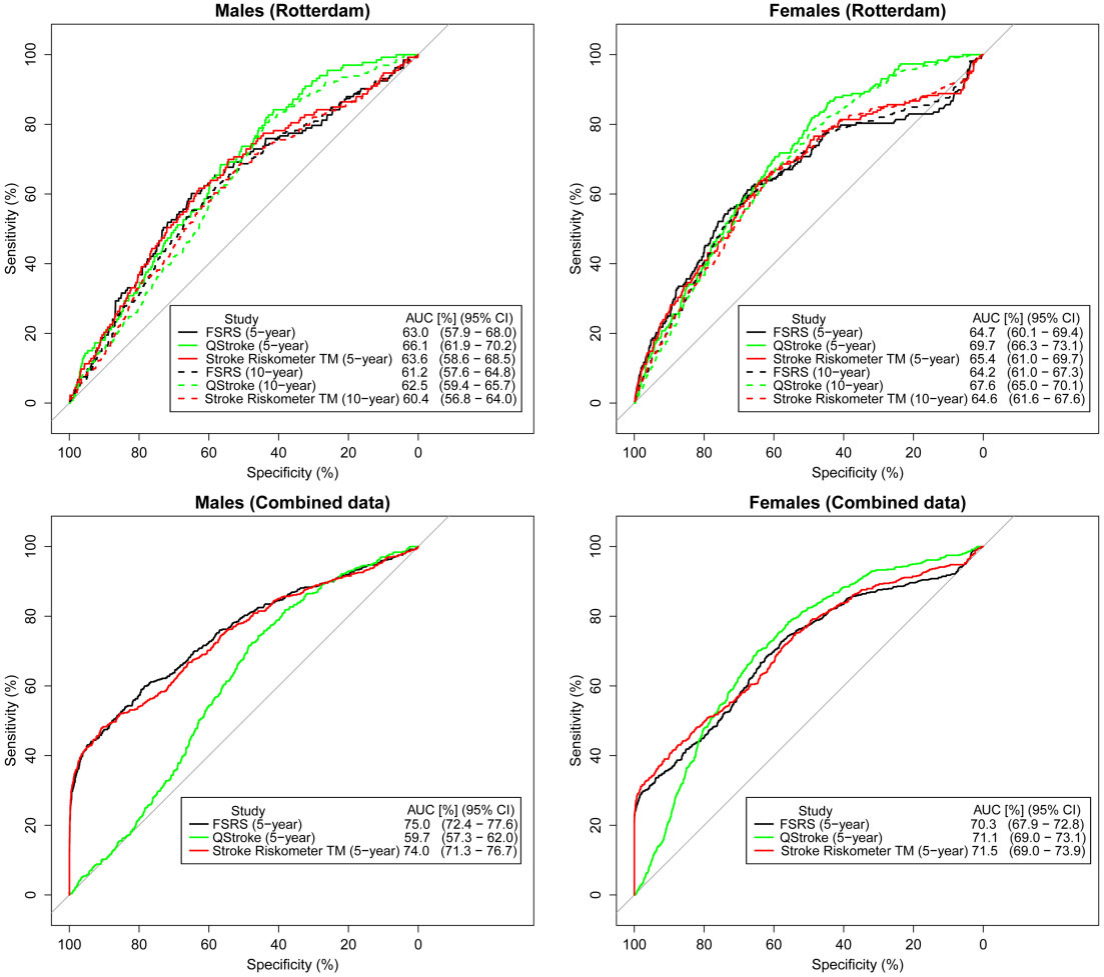
Receiver-operating characteristic (ROC) curves for Framingham Stroke Risk Score (FSRS) (black), Stroke Riskometer™ (red) and QStroke (green) algorithms for 5 and 10-year risks.

All three algorithms showed poor discriminative ability across each cohort (C-statistic range 0·50–0·53, D-statistic <0·05, Table 3). The ROC curves (Fig. 2) show that the FSRS and Stroke Riskometer™ algorithms behaved similarly for 5-year and 10-year risk scores for males and females, with area under the ROC curves ranging between 61% and 66% in the Rotterdam cohort (Fig. 2, Table 3). The QStroke algorithm outperformed the FSRS and Stroke Riskometer™ algorithms (Table 3).

When all three data sets (ARCOS, Russia and Rotterdam) were combined the Stroke Riskometer™ and FSRS algorithms had higher five-year AUROC values for males [FSRS AUROC = 75·0 (95% CI 72·5%–77·6%), Stroke Riskometer™ AUROC = 74·0% (95% CI 71·3%–76·7%)], for both FSRS and Stroke Riskometer™ C-statistic = 0·56 and D-statistic = 0·12) and females (FSRS AUROC = 70·3% (95% 67·9%–72·8%), Stroke Riskometer™ AUROC = 71·5% (95% CI 69·0%–73·9%), for both FSRS and Stroke Riskometer™ C-statistic = 0·54 and D-statistic = 0·08). There was no difference in the AUROC between the FSRS and Stroke Riskometer™ AUROC (DeLong's for correlated ROC curves; males *P* = 0·013, females *P* = 0·140). AUROC for QStroke were considerably lower (males AUROC = 59·7% (95% CI 57·3%–62·0 %), C-statistic = 0·52, D-statistic = 0·04 and for females AUROC = 71·1% (95% CI 69·0%–73·1%), C-statistic = 0·54, D-statistic = 0·08) (Fig. 2). A statistically significant difference in the AUROC between the QStroke and Stroke Riskometer™ was observed (DeLong's test for correlated ROC curves; males *P* < 0·0001, females *P* = 0·779).

### Classification, sensitivity and specificity

Mean predicted stroke risk scores were on average higher in the group of observed stroke outcomes compared to individuals with no stroke outcome (Supplementary Fig. S1). Sensitivity and specificity was calculated for FSRS, Stroke Riskometer™ and QStroke predicted risk scores, which reached accuracy threshold of 50%, 70%, 80%, 85% and 90% (Table 4). The predicted risk scores were then categorized into ‘High’ risk (based on reaching 80% accuracy and >80% specificity, Table 4) and ‘Low’ otherwise, which were compared against each other (Table 5). Sensitivity for QStroke in males was low (10·6% for accuracy = 80%) compared to FSRS and Stroke Riskometer™ which had a sensitivity = 53·9% (FRSR) and 52·3% (Stroke Riskometer™) for accuracy = 80%.

**Table 4:** Performance of risk score algorithms (Framingham Stroke Risk Score (FSRS), Stroke Riskometer™ and QStroke) across three validation cohorts (ARCOS, RUSSIA and ROTTERDAM) combined across different thresholds meeting 50%, 70%, 80%, 85% and 90% accuracy

Algorithm	Subset	5-year risk				10-year risk			
		Threshold [Accuracy (%)]	Number classified as high risk (%)	Sensitivity (%)	Specificity (%)	Threshold [Accuracy (%)]	Number classified as high risk (%)	Sensitivity (%)	Specificity (%)
FSRS	Males	4·25 (50)	2202 (58·6)	82·49	45·02	11·3 (50)	2200 (58·51)	82·49	45·08
		8·6 (70)	1273 (33·9)	63·38	70·61	22 (70)	1270 (33·78)	63·38	70·70
		13 (80)	807 (21·5)	53·92	83·45	32 (80)	798 (21·22)	53·72	83·73
		18 (85)	538 (14·3)	46·28	90·56	42 (85)	542 (14·41)	46·48	90·47
		30 (90)	245 (6·5)	35·21	97·85	65 (90)	227 (6·04)	33·80	98·19
	Females	3·2 (50)	3190 (55·6)	78·89	47·04	8 (50)	3185 (55·52)	78·89	47·16
		9·5 (70)	1801 (31·4)	55·28	71·30	23 (70)	1770 (30·85)	54·44	71·84
		19·5 (80)	1055 (18·4)	42·04	84·33	42 (80)	1064 (18·55)	42·38	84·19
		28 (85)	677 (11·8)	35·51	90·92	57 (85)	672 (11·71)	35·51	91·02
		42 (90)	361 (6·3)	30·15	96·44	79 (90)	304 (5·30)	29·65	97·49
Stroke Riskometer™	Males	5·7 (50)	2184 (58·1)	81·49	45·42	13 (50)	2223 (59·12)	82·90	44·50
		14·5 (70)	1218 (32·4)	59·56	71·71	30 (70)	1225 (32·58)	61·37	71·81
		21·5 (80)	770 (20·5)	52·31	84·37	43 (80)	789 (20·98)	52·52	83·82
		27 (85)	515 (13·7)	46·48	91·30	55 (85)	514 (13·67)	46·08	91·27
		45 (90)	188 (5·0)	31·79	99·08	72 (90)	279 (7·42)	38·43	97·30
	Females	4·5 (50)	3212 (56·0)	80·07	46·75	10 (50)	3219 (56·12)	80·40	46·67
		13·5 (70)	1803 (31·4)	55·61	71·33	27 (70)	1787 (31·15)	56·28	71·70
		22 (80)	1069 (18·6)	44·89	84·34	45 (80)	1080 (18·83)	45·06	84·17
		29 (85)	734 (12·8)	39·03	90·20	57 (85)	745 (12·99)	39·20	90·00
		45 (90)	336 (5·9)	31·66	97·08	77 (90)	373 (6·50)	33·00	96·52
QStroke	Males	2·5 (50)	2130 (56·6)	73·44	45·91	6·7 (50)	2090 (55·59)	72·43	46·98
		5·3 (70)	910 (24·2)	26·76	76·19	13·5 (70)	912 (24·25)	26·96	76·16
		8·6 (80)	357 (9·5)	10·06	90·59	22 (80)	325 (8·64)	8·85	91·39
		14 (85)	77 (2·0)	2·21	97·98	33 (85)	78 (2·07)	2·21	97·95
	Females	2·4 (50)	3270 (57·0)	84·25	46·13	6·3 (50)	3269 (56·99)	84·25	46·15
		7·7 (70)	1806 (31·5)	59·13	71·68	19 (70)	1822 (31·76)	59·63	71·43
		23 (80)	978 (17·1)	35·85	85·08	48 (80)	1012 (17·64)	36·68	84·52
		70 (85)	391 (6·8)	12·56	93·80	95 (85)	422 (7·36)	13·74	93·33

**Table 5:** Comparing the scoring of the three risk score algorithms as ‘High’ or ‘Low’ risk for Framingham Stroke Risk Score (FSRS), Stroke Riskometer™ and the Qstroke algorithm across all validation cohorts (ARCOS, RUSSIA and ROTTERDAM). Thresholds for ‘High’ risk in each algorithm for males and females was selected for 80% accuracy and >80% specificity (Table 4)

Algorithm	Comparison	Subset	Number of patients (%)					
			RUSSIA		ARCOS		ROTTERDAM	
Stroke Riskometer™ vs. FSRS			5-year risk	10-year risk	5-year risk	10-year risk	5-year risk	10-year risk
Low risk on Stroke Riskometer™	Low risk on FSRS	Males	20 (4·16%)		0 (0·00%)		2410 (78·63%)	2522 (82·28%)
High risk on Stroke Riskometer™	Low risk on FSRS		155 (32·22%)		3 (1·40%)		275 (8·97%)	163 (5·32%)
Low risk on Stroke Riskometer™	High risk on FSRS		0 (0·00%)		1 (0·47%)		17 (0·55%)	6 (0·20%)
High risk on Stroke Riskometer™	High risk on FSRS		306 (63·62%)		210 (98·13%)		363 (11·84%)	374 (12·20%)
Low risk on Stroke Riskometer™	Low risk on FSRS	Females	190 (21·18%)		3 (1·40%)		4114 (88·51%)	4188 (90·10%)
High risk on Stroke Riskometer™	Low risk on FSRS		1 (0·11%)		6 (2·80%)		194 (4·17%)	119 (2·56%)
Low risk on Stroke Riskometer™	High risk on FSRS		0 (0·00%)		0 (0·00%)		3 (0·00%)	0 (0·00%)
High risk on Stroke Riskometer™	High risk on FSRS		703 (78·37%)		185 (86·45%)		337 (7·25%)	341 (7·34%)

Stroke Riskometer™ vs. QStroke			RUSSIA		ARCOS		ROTTERDAM	
Low risk on Stroke Riskometer™	Low risk on QStroke	Males	20 (4·16%)		0 (0·00%)		2258 (73·67%)	2346 (76·54%)
High risk on Stroke Riskometer™	Low risk on QStroke		439 (91·27%)		208 (97·20%)		376 (12·27%)	279 (9·10%)
Low risk on Stroke Riskometer™	High risk on QStroke		0 (0·00%)		1 (0·47%)		47 (5·51%)	182 (5·94%)
High risk on Stroke Riskometer™	High risk on QStroke		22 (4·57%)		5 (2·34%)		262 (8·55%)	258 (8·42%)
Low risk on Stroke Riskometer™	Low risk on QStroke	Females	190 (21·18%)		3 (1·40%)		3505 (75·41%)	3557 (76·53%)
High risk on Stroke Riskometer™	Low risk on QStroke		686 (76·48%)		103 (48·13%)		168 (3·61%)	116 (2·50%)
Low risk on Stroke Riskometer™	High risk on QStroke		0 (0·00%)		0 (0·00%)		612 (13·17%)	631 (13·58%)
High risk on Stroke Riskometer™	High risk on QStroke		18 (2·01%)		88 (41·12%)		363 (7·81%)	344 (7·40%)

For FSRS: Male 5-year = 13·0%, Male 10-year = 32·0%, Female 5-year = 19·5%, Female 10-year = 42·0%. For Stroke Riskometer™: Male 5-year = 21·5%, Male 10-year = 43·0%, Female 5-year = 22·0%, Female 10-year = 45·0%. For QStroke: Male 5-year = 8·6%, Male 10-year = 22·0%, Female 5-year = 23·0%, Female 10-year = 48·0%.

In the Russian database we observed that both the Stroke Riskometer™ and FSRS algorithms classified most participants as high risk (63·6% five-year risk in males and 78·4% five-year risk in females). As ARCOS had all stroke events we would expect these to predominately to be categorized as ‘High risk’ this is observed for FSRS and Stroke Riskometer™ (males = 98·1% and females = 86·4%). A very high proportion of individuals in the ARCOS data set were classified as high risk for Stroke Riskometer™ but low risk on QStroke (males = 97·2% and females = 48·1% for five-year risk). A high proportion of females in the Rotterdam study were categorized as low-risk for Stroke Riskometer™ and high-risk for QStroke (13·2% for five-year risk in females) compared to 5·5% of males classified as low-risk for Stroke Riskometer™ and high-risk for QStroke (Table 5).

### Calibration

**Figure 3:**
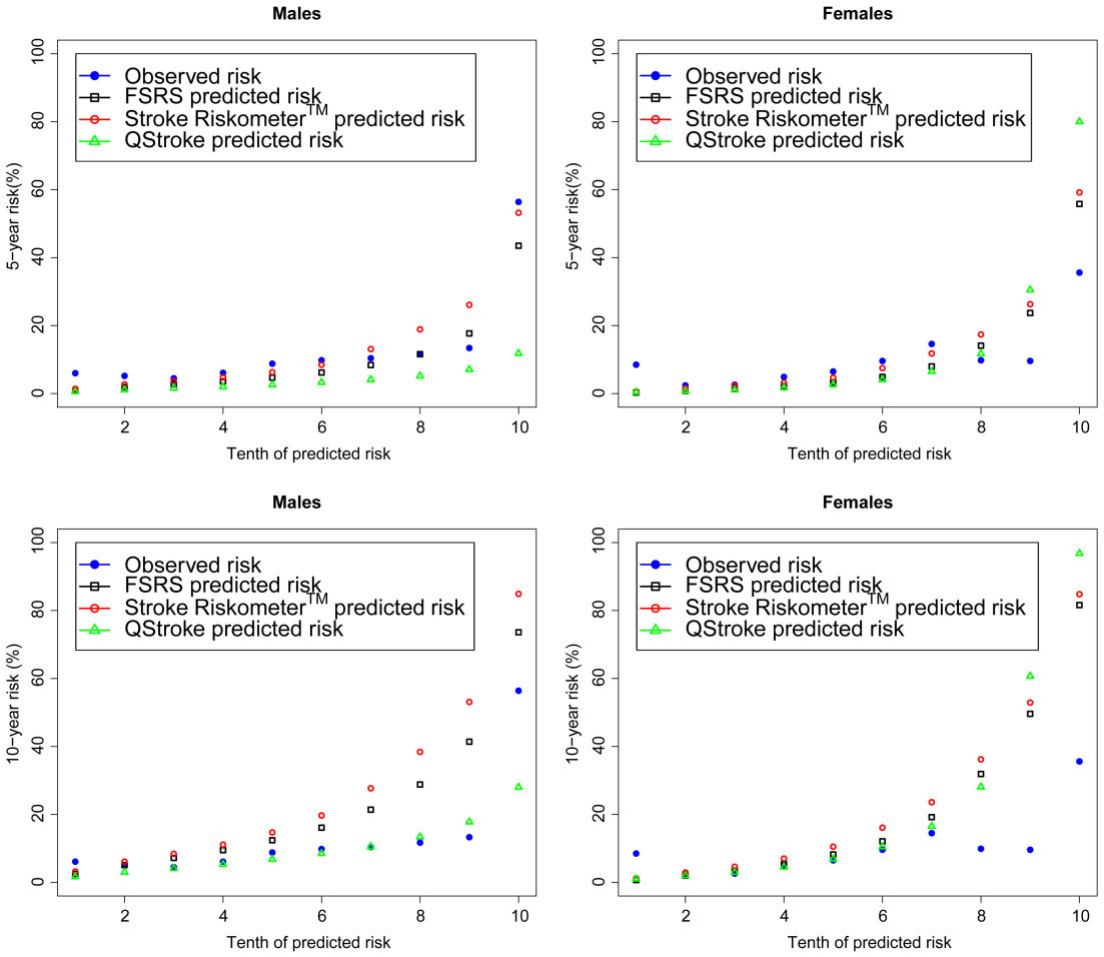
Mean predicted risk (%) vs. observed stroke events in deciles of predicted risk for Framingham Stroke Risk Score (FSRS) (black), Stroke Riskometer™ (red) and QStroke (green) algorithms.

Calibration plots of the predicted risk scores against the observed event for each tenth of predicted risk, separately for males and females, are shown in Fig. 3 (all data sources) and Supplementary Fig. S2 (Russian and Rotterdam cohorts). The Russian cohort illustrated that the QStroke algorithm was better calibrated for the data set of all three algorithms, for a database with few strokes (Supplementary Fig. S2). An improved calibration for the FSRS and Stroke Riskometer™ algorithms compared to QStroke was observed for the Rotterdam data set, particularly for females. The QStroke algorithm was shown to over-estimate stroke risk in females whilst FSRS and Stroke Riskometer™ over-estimated stroke risk in males (Supplementary Fig. S2). Visual assessment of five-year risk scores from the combined data (ARCOS, Russia and Rotterdam) highlighted that the Stroke Riskometer™ algorithm was better calibrated compared to QStroke, especially for females (Fig. 3). All predicted risk scores were not well calibrated to our data sets (Table 6, H-L tests *P* < 0·006).

**Table 6:** Performance of the goodness-of-fit of each algorithm reported as the Hosmer-Lemeshow calibration statistic for Framingham Stroke Risk Score (FSRS), Stroke Riskometer™ and QStroke against observed stroke events at 5-years for the Russian and 5-years and 10-years for the Rotterdam and combined (ARCOS, Russia and Rotterdam data set)

Data	Risk score	Subset	Hosmer-Lemeshow Test	
			5-year risk	10-year risk
RUSSIA	FSRS	Females	χ2 = 58·12, *P* ≤ 0·0001	
		Males	χ2 = 133·65, *P* ≤ 0·0001	
	Stroke Riskometer™	Females	χ2 = 321·92, *P* ≤ 0·0001	
		Males	χ2 = 36·84, *P* ≤ 0·0001	
	QStroke	Females	χ2 = 3·33, *P* = 0·912	
		Males	χ2 = 318·81, *P* ≤ 0·0001	
Rotterdam	FSRS	Females	χ2 = 69·95, *P* ≤ 0·0001	χ2 = 222·02, *P* ≤ 0·0001
		Males	χ2 = 100·58, *P* ≤ 0·0001	χ2 = 356·01, *P* ≤ 0·0001
	Stroke Riskometer™	Females	χ2 = 298·95, *P* ≤ 0·0001	χ2 = 588·20, *P* ≤ 0·0001
		Males	χ2 = 2247·03, *P* ≤ 0·0001	χ2 = 20 297·53, *P* ≤ 0·0001
	QStroke	Females	χ2 = 21·68, *P* = 0·006	χ2 = 70·10, *P* ≤ 0·0001
		Males	χ2 = 796·93, *P* ≤ 0·0001	χ2 = 949·04, *P* ≤ 0·0001
Combined	FSRS	Females	χ2 = 196·70, *P* ≤ 0·0001	χ2 = 304·91, *P* ≤ 0·0001
		Males	χ2 = 153·78, *P* ≤ 0·0001	χ2 = 726·04, *P* ≤ 0·0001
	Stroke Riskometer™	Females	χ2 = 547·29, *P* ≤ 0·0001	χ2 = 1 811·14, *P* ≤ 0·0001
		Males	χ2 = 1699·96, *P* ≤ 0·0001	χ2 = 11 552·55, *P* ≤ 0·0001
	QStroke	Females	χ2 = 1441·52, *P* ≤ 0·0001	χ2 = 270·42, *P* ≤ 0·0001
		Males	χ2 = 1587·38, *P* ≤ 0·0001	χ2 = 1 822·10, *P* ≤ 0·0001

## Discussion

The Stroke Riskometer™ is comparable in performance to two widely used stroke risk scoring systems. The variation found in our study may be due to several factors. The higher R^2^ values for ARCOS are explained by the high number of stroke outcome data available. Many variables required for the QStroke algorithm were not available within the ARCOS data set (such as rheumatoid arthritis, chronic kidney disease, Table 2) therefore it is likely that the QStroke risk scores we observed under-estimate stroke risk, particularly amongst males in ARCOS. A large proportion of females in ARCOS were classified as high risk in both Stroke Riskometer™ and QStroke scoring (>50%, Table 5). Conversely, the Rotterdam study had a more complete data set of variables required for the QStroke algorithm calculation (Table 2), however this appears to have led to over-estimation of the stroke risk amongst females (Fig. 1, Supplementary Fig. S2C) and an inconsistency across predicted risk scores with 13% categorized as low-risk for Stroke Riskometer™ and high-risk for QStroke (Table 5). Sensitivity was low for the QStroke risk scores generated for males (10·6% for 5-year and 8·9% for 10-year risk scores) and females (35·9% for 5-year and 36·7% for 10-year risk scores, Table 4), when specificity was high (= 80%, Table 4) compared to the sensitivity for FSRS and Stroke Riskometer™ for males (53% for 5-year and 10-year risk scores) and females (42% for 5-year and 10-year risk scores, Table 4) for FSRS, and (45% for 5-year and 10-year risk scores, Table 4 for Stroke Riskometer™, when specificity was high (= 80%, Table 4). The developers of QStroke have previously highlighted that their algorithm over-predicts stroke risk in females 8. It should also be noted that QStroke was developed for predicting ischaemic stroke specifically, and not for predicting any type of stroke as developed for Stroke Riskometer™ and FSRS.

Whilst the discriminative abilities of all three algorithms across all data sets appeared to be comparable, they were also very low (C-statistic ranging from 0·51–0·56, D-statistic ranging from 0·01–0·12). H-L calibration statistics illustrated that all of the predicted risk scores did not align well to observed event data, *P* < 0·006. This may be due to the QStroke risk score algorithms being developed from UK-based data and while the data sets being utilized here are predominately European, they were not UK-based individuals. The FSRS has been externally validated in several different European cohorts but with inconsistent result, some studies attaining appropriate levels of discrimination but over-estimation of risk of stroke 51, however other studies have shown the FSRS has poor discrimination and under-estimates stroke risk 52. QStroke was recently created and validated in a subset of the British cohort data used to develop their algorithm and showed good levels of discrimination; however the authors did acknowledge a tendency to overestimate female stroke risk 8. In a large cohort of black and white adults the FSRS overestimated observed stroke rates, particularly in certain ethnic subgroups where the FSRS suggested there should be approximately twice as many strokes occurring than was detected 53.

This indicates that there is still a need to improve current stroke risk scoring systems to more accurately predict stroke risk across different populations/countries. We have shown that there is a level of overlap in the variables considered in these algorithms, however it may be that the weights assigned to each risk factor need to be generated to be country/or ethnic-specific as some risk factors may hold more importance in some groups compared to others 54. It is also likely that there are further unknown stroke risk factors that still need to be identified and included in a stroke prediction assessment tool. For one such example we refer to the recent evidence from Yusuf *et al*. 55 that populations from low to middle-income countries are at highest risk of cardiovascular events have the lowest risk factor burden 55, suggesting that the major ‘missing piece in the equation’ of the effective CVD prevention is the impaired ability of resource-limited health systems to effectively identify and modify cardiovascular risk. It is our expectation that the Stroke Riskometer™ will be further developed to account for these factors (we are currently collecting data on country) such that in future iterations of the Stroke Riskometer™ we hope to refine the algorithm to be able to provide country and ethnic specific-stroke risk prediction estimates, using both current research such as Yusuf *et al*. 55 and data collected from the current Stroke Riskometer™ App to improve overall predictability and applicability of our algorithm across all populations. Furthermore, an algorithm for all major noncommunicable disease, such as stroke, ischaemic heart disease (IHD), dementia and diabetes mellitus that share common risk factors, should be developed and validated in different populations. The main weakness of this validation study was that analyses were restricted due to the lack of currently available data on the variables shown to be important determinants of stroke.

The Stroke Riskometer™ availability on a portable device (smartphone) with constant proximity to the user, enables individuals to assess their own risk of stroke in the privacy and comfort anytime, anywhere. Unlike web-based versions, no internet connection required to use the app or access its information. In addition, the app offers a higher level of interactivity via sending direct reminders to the smartphone that is always on hand when needed. Moreover, the availability of the app on the smartphone app stores that has global reach, and vast consumer base of various age groups allows wide range of consumers to benefit from the stroke risk assessment tool and allows the crowdsourcing of large research database. Finally, users who are at increased (even slightly increased) risk are provided with ways to reduce their risk of stroke according to their individual risk profile and recommended to seek medical attention. This could rapidly transform epidemiologic research and monitoring of health status of individuals, especially in the area of chronic NCD 17.

Current risk scores will inevitably become outdated with improvements in clinical outcomes and data recording and changes in population demographics 56. With the Lite version of the Stroke Riskometer™ being made freely available globally on both iOS and Android smartphones and users invited to partake in a large-scale study we will have the potential to amass a large database. Ethical approval for the study has been received. Anonymous data from individuals who consent to participate in the study will be collected and securely stored at study coordinating centre (AUT University, NZ). The aim of these planned epidemiological studies based on the Stroke Riskometer™ will be to generate a global, population-specific stroke and NCD risk scoring system. We will further assess the Stroke Riskometer™ in a cohort study to establish the efficiency of the algorithm and assess if the new collections of recommendations are useful for motivating users to actively reduce their risk of stroke.

## Supporting Information

**Fig. S1.** Bar plots of mean predicted risk scores for observed stroke and nonstroke events at (A) 5-years and (B) 10-years for FSRS (black), Stroke Riskometer™ (red) and QStroke (green) algorithms across the (1A) Rotterdam and (1B) combined [ARCOS, Russia and Rotterdam] data sets for males (left) and females (right). **Fig. S2.** Mean predicted risk (%) vs. observed stroke events in deciles of predicted risk for Framingham Stroke Risk Score (FSRS) (black), Stroke Riskometer™ (red) and QStroke (green) algorithms for males (left) and females (right) for (A) 5-year predicted risks for the Russian data set, (B) 5-year predicted risks for the Rotterdam data set and (C) 10-year predicted risks for the Rotterdam data set.

## References

[b1] Lozano R, Naghavi M, Foreman K (2012). Global and regional mortality from 235 causes of death for 20 age groups in 1990 and 2010: a systematic analysis for the global burden of disease study 2010. Lancet.

[b2] Feigin VL, Forouzanfar MH, Krishnamurthi R (2014). Global and regional burden of stroke during 1990–2010: findings from the global burden of disease study 2010. Lancet.

[b3] Bos MJ, Koudstaal PJ, Hofman A, Ikram MA (2014). Modifiable etiological factors and the burden of stroke from the rotterdam study: a population-based cohort study. PLoS Med.

[b4] O'Donnell MJ, Xavier D, Liu L (2010). Risk factors for ischaemic and intracerebral haemorrhagic stroke in 22 countries (the interstroke study): a case-control study. Lancet.

[b5] Danaei G, Finucane MM, Lu Y (2011). National, regional, and global trends in fasting plasma glucose and diabetes prevalence since 1980: systematic analysis of health examination surveys and epidemiological studies with 370 country-years and 2¬Σ7 million participants. Lancet.

[b6] Ng M, Fleming T, Robinson M (2014). Global, regional, and national prevalence of overweight and obesity in children and adults during 1980–2013: a systematic analysis for the global burden of disease study 2013. Lancet.

[b7] Wolf PA, D'Agostino RB, Belanger AJ, Kannel WB (1991). Probability of stroke: a risk profile from the framingham study. Stroke.

[b8] Hippisley-Cox J, Coupland C, Brindle P (2013). Derivation and validation of qstroke score for predicting risk of ischaemic stroke in primary care and comparison with other risk scores: a prospective open cohort study. BMJ.

[b9] Rose G (1981). Strategy of prevention: lessons from cardiovascular disease. Br Med J (Clin Res Ed).

[b10] Brindle P, Emberson J, Lampe F (2003). Predictive accuracy of the framingham coronary risk score in british men: prospective cohort study. BMJ.

[b11] Cooney MT, Dudina A, Whincup P (2009). Re-evaluating the rose approach: comparative benefits of the population and high-risk preventive strategies. Eur J Cardiovasc Prev Rehabil.

[b12] Dalton AR, Soljak M, Samarasundera E, Millett C, Majeed A (2013). Prevalence of cardiovascular disease risk amongst the population eligible for the nhs health check programme. Eur J Prev Cardiol.

[b13] Jones SP, Jenkinson AJ, Leathley MJ, Watkins CL (2010). Stroke knowledge and awareness: an integrative review of the evidence. Age Ageing.

[b14] Hickey A, O'Hanlon A, McGee H (2009). Stroke awareness in the general population: knowledge of stroke risk factors and warning signs in older adults. BMC Geriatr.

[b15] Roger VL, Go AS, Lloyd-Jones DM (2011). Heart disease and stroke statistics – 2011 update: a report from the american heart association. Circulation.

[b16] Cooney MT, Dudina A, D'Agostino R, Graham IM (2010). Cardiovascular risk-estimation systems in primary prevention: do they differ? Do they make a difference? Can we see the future?. Circulation.

[b17] Brouillette RM, Foil H, Fontenot S (2013). Feasibility, reliability, and validity of a smartphone based application for the assessment of cognitive function in the elderly. PLoS ONE.

[b18] Leung R, Tang C, Haddad S, McGrenere J, Graf P, Ingriany V (2012). How older adults learn to use mobile devices: survey and field investigations. ACM Trans Access Comput.

[b19] Arab F, Malik Y, Abdulrazak B (2013). Evaluation of phonage: an adapted smartphone interface for elderly people. Lect Notes Comput Sci.

[b20] Be he@lthy, be mobile (2014). http://www.itu.int/en/ITU-D/ICT-Applications/eHEALTH/Be_healthy/Pages/Be_Healthy.aspx.

[b21] Hill S, Spink J, Cadilhac D (2010). Absolute risk representation in cardiovascular disease prevention: comprehension and preferences of health care consumers and general practitioners involved in a focus group study. BMC Public Health.

[b22] Fagerlin A, Zikmund-Fisher BJ, Ubel PA (2011). Helping patients decide: ten steps to better risk communication. J Natl Cancer Inst.

[b23] Goldstein LB, Bushnell CD, Adams RJ (2011). Guidelines for the primary prevention of stroke: a guideline for healthcare professionals from the american heart association/american stroke association.[erratum appears in stroke. 2011 feb;42(2):E26]. Stroke.

[b24] Eckel RH, Jakicic JM, Ard JD (2014). 2013 aha/acc guideline on lifestyle management to reduce cardiovascular risk: a report of the american college of cardiology/american heart association task force on practice guidelines. J Am Coll Cardiol.

[b25] Furie KL, Kasner SE, Adams RJ (2011). Guidelines for the prevention of stroke in patients with stroke or transient ischemic attack: a guideline for healthcare professionals from the american heart association/american stroke association. Stroke.

[b26] Graham I, Atar D, Borch-Johnsen K (2007). European guidelines on cardiovascular disease prevention in clinical practice: executive summary: fourth joint task force of the european society of cardiology and other societies on cardiovascular disease prevention in clinical practice (constituted by representatives of nine societies and by invited experts). Eur Heart J.

[b27] Perk J, De Backer G, Gohlke H (2012). European guidelines on cardiovascular disease prevention in clinical practice (version 2012). The fifth joint task force of the european society of cardiology and other societies on cardiovascular disease prevention in clinical practice (constituted by representatives of nine societies and by invited experts).[erratum appears in eur heart j. 2012 sep;33(17):2126]. Eur Heart J.

[b28] WHO (2007). http://www.who.int/cardiovascular_diseases/resources/publications/en/index.html.

[b29] Feigin VL, Norrving B (2014). A new paradigm for primary prevention strategy in people with elevated risk of stroke. Int J Stroke.

[b30] Krishnamurthi R, Jones A, Barber A (2014). Methodology of a population-based stroke and tia incidence and outcomes study: the auckland regional community stroke study (arcos iv) 2011–2012. Int J Stroke.

[b31] Hofman A, Darwish Murad S, van Duijn CM (2013). The rotterdam study: 2014 objectives and design update. Eur J Epidemiol.

[b32] Clark TG, Altman DG, De Stavola BL (2002). Quantification of the completeness of follow-up. Lancet.

[b33] Hatano S (1976). Experience from a multicentre stroke register: a preliminary report. Bull World Health Organ.

[b34] WHO (2013). Geneva World Health Organization.

[b35] van Asch CJJ, Luitse MJ, Rinkel GJ, van der Tweel I, Algra A, Klijn CJ (2010). Incidence, case fatality, and functional outcome of intracerebral haemorrhage over time, according to age, sex, and ethnic origin: a systematic review and meta-analysis. Lancet Neurol.

[b36] Shinton R, Sagar G, Beevers G (1993). The relation of alcohol consumption to cardiovascular risk factors and stroke. The west birmingham stroke project. J Neurol Neurosurg Psychiatry.

[b37] Bazzano LA, Gu D, Reynolds K (2007). Alcohol consumption and risk for stroke among chinese men. Ann Neurol.

[b38] Mvundura M, McGruder H, Khoury MJ, Valdez R, Yoon PW (2010). Family history as a risk factor for early-onset stroke/transient ischemic attack among adults in the united states. Public Health Genomics.

[b39] Liao D, Myers R, Hunt S (1997). Familial history of stroke and stroke risk: the family heart study. Stroke.

[b40] Kayaba K (2008). Family history of stroke: an old and still unproven risk factor. Hypertens Res.

[b41] Cook NR, Paynter NP, Eaton CB (2012). Comparison of the framingham and reynolds risk scores for global cardiovascular risk prediction in the multiethnic women's health initiative. Circulation.

[b42] Pendlebury ST, Rothwell PM (2009). Risk of recurrent stroke, other vascular events and dementia after transient ischaemic attack and stroke. Cerebrovasc Dis.

[b43] Ferrucci L, Guralnik JM, Salive ME (1996). Cognitive impairment and risk of stroke in the older population. J Am Geriatr Soc.

[b44] Chen YH, Kang JH, Lin HC (2011). Patients with traumatic brain injury: population-based study suggests increased risk of stroke. Stroke.

[b45] De Koning L, Merchant AT, Pogue J, Anand SS (2007). Waist circumference and waist-to-hip ratio as predictors of cardiovascular events: meta-regression analysis of prospective studies. Eur Heart J.

[b46] Asplund K, Karvanen J, Giampaoli S (2009). Relative risks for stroke by age, sex, and population based on follow-up of 18 european populations in the morgam project. Stroke.

[b47] D'Agostino RB, Vasan RS, Pencina MJ (2008). General cardiovascular risk profile for use in primary care: the framingham heart study. Circulation.

[b48] Chang M, Hahn RA, Teutsch SM, Hutwagner LC (2001). Multiple risk factors and population attributable risk for ischemic heart disease mortality in the united states, 1971–1992. J Clin Epidemiol.

[b49] Pennells L, Kaptoge S, White IR, Thompson SG, Wood AM, Factors ER (2014). Assessing risk prediction models using individual participant data from multiple studies. Am J Epidemiol.

[b50] R Development Core Team (2013).

[b51] Bineau S, Dufouil C, Helmer C (2009). Framingham stroke risk function in a large population-based cohort of elderly people: the 3c study. Stroke.

[b52] Majed B, Tafflet M, Kee F (2013). External validation of the 2008 framingham cardiovascular risk equation for chd and stroke events in a european population of middle-aged men. The prime study. Prev Med.

[b53] McClure LA, Kleindorfer DO, Kissela BM, Cushman M, Soliman EZ, Howard G (2014). Assessing the performance of the framingham stroke risk score in the reasons for geographic and racial differences in stroke cohort. Stroke.

[b54] Arts EE, Popa C, Den Broeder AA (2014). Performance of four current risk algorithms in predicting cardiovascular events in patients with early rheumatoid arthritis. Ann Rheum Dis.

[b55] Yusuf S, Rangarajan S, Teo K (2014). Cardiovascular risk and events in 17 low-, middle-, and high-income countries. N Engl J Med.

[b56] Collins GS, Altman DG (2010). An independent and external validation of qrisk2 cardiovascular disease risk score: a prospective open cohort study. BMJ.

