# Supplementary material for: The Stroke Riskometer™ App: Validation of a data collection tool and stroke risk predictor
Source: Int J Stroke. 2014 Dec 10;10(2):231–44. doi: 10.1111/ijs.12411 (PMC4335600; doi:10.1111/ijs.12411)

SUPPLEMENTARY MATERIALS

Supplementary Figure 1A

(A)

Supplementary Figure 1A

(B)

(A)

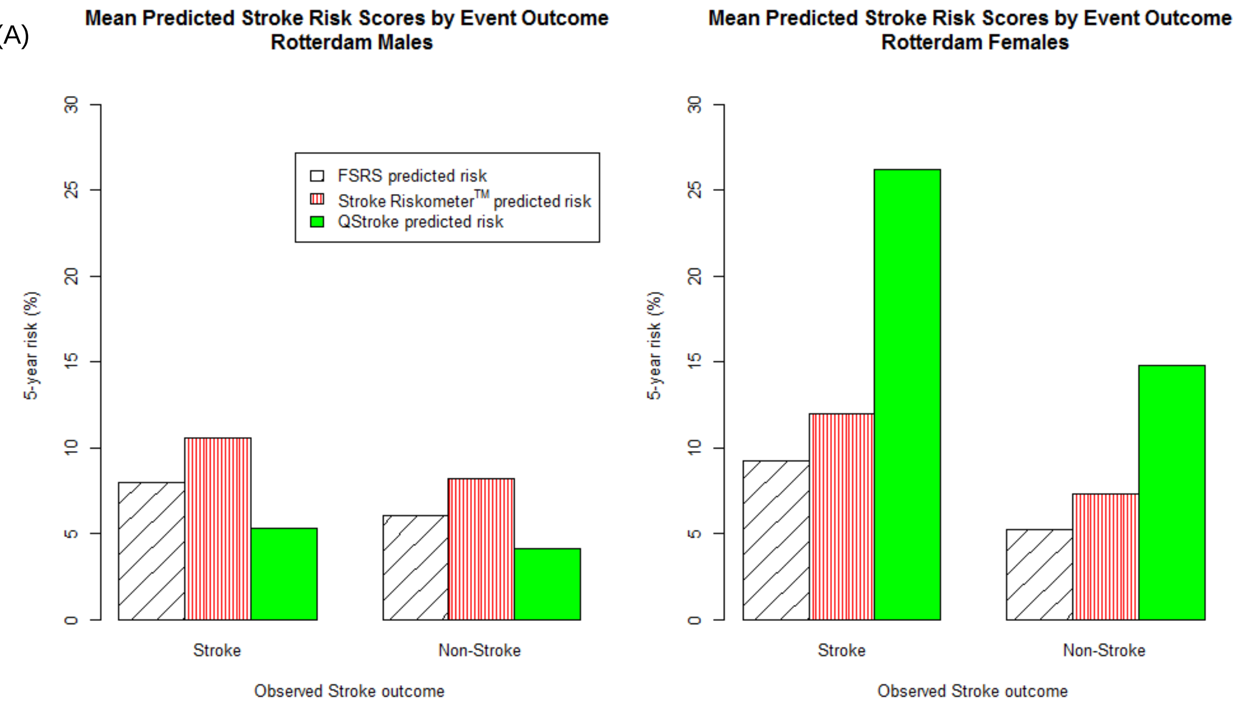

(B)

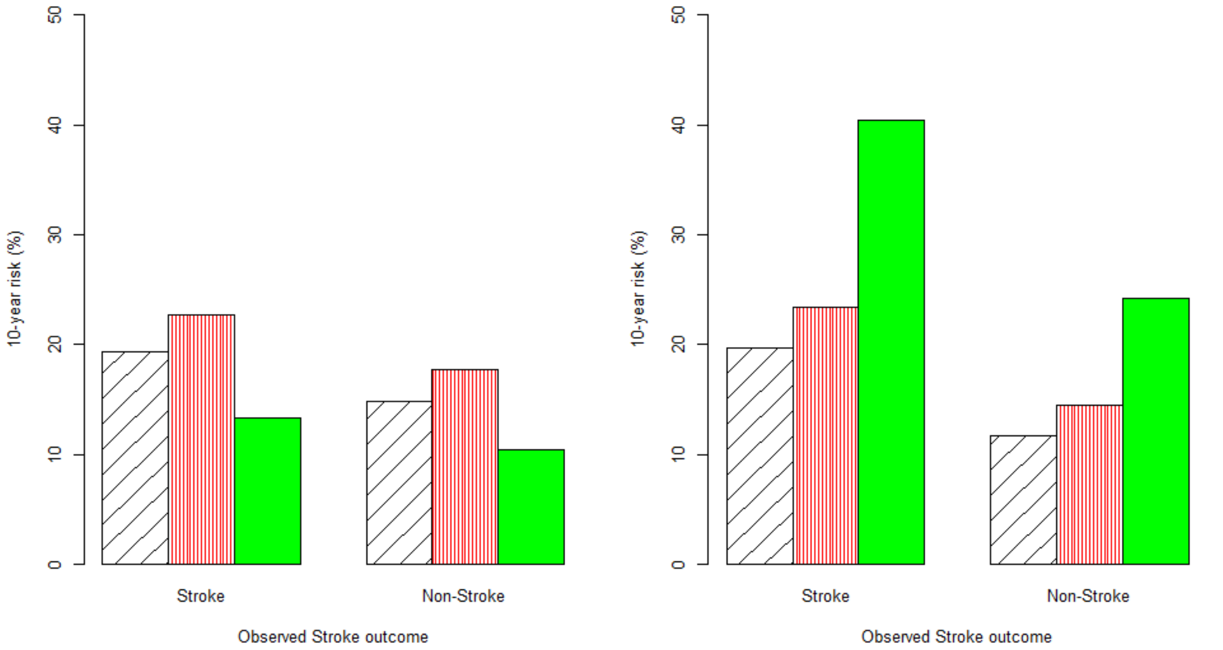

Supplementary Figure 1B

(A)

Supplementary Figure 1B

(A)

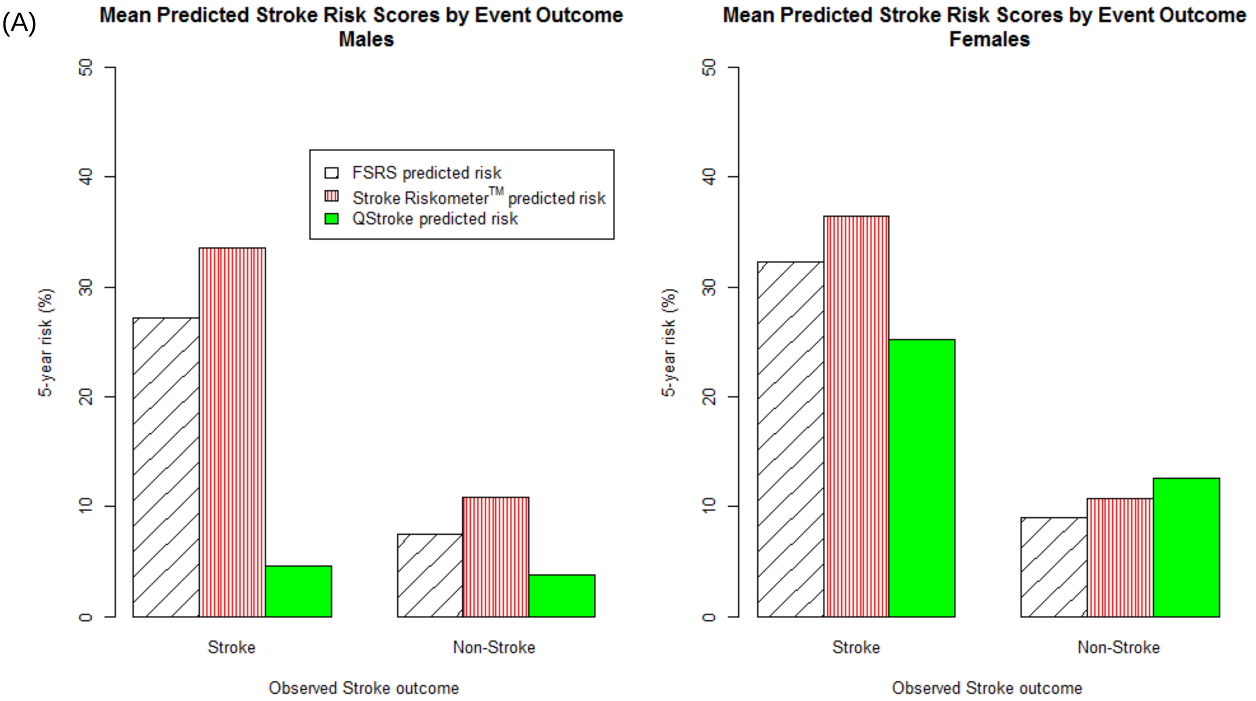

(B)

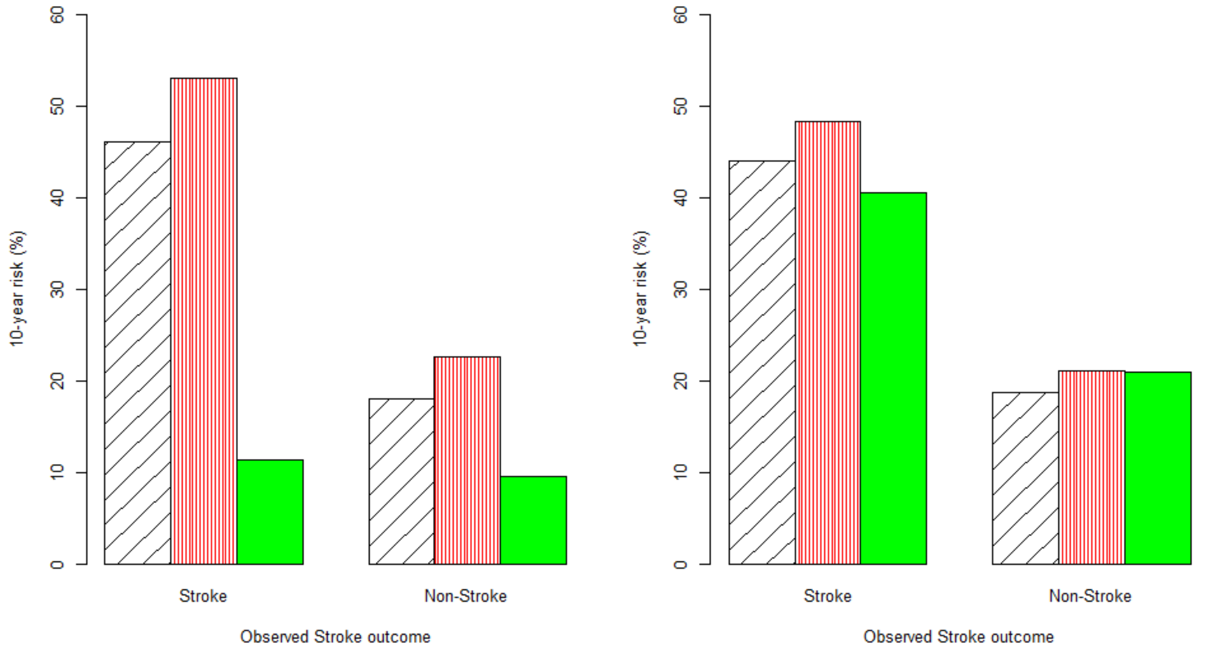

Supplementary Figure 2

(A)

Supplementary Figure 2

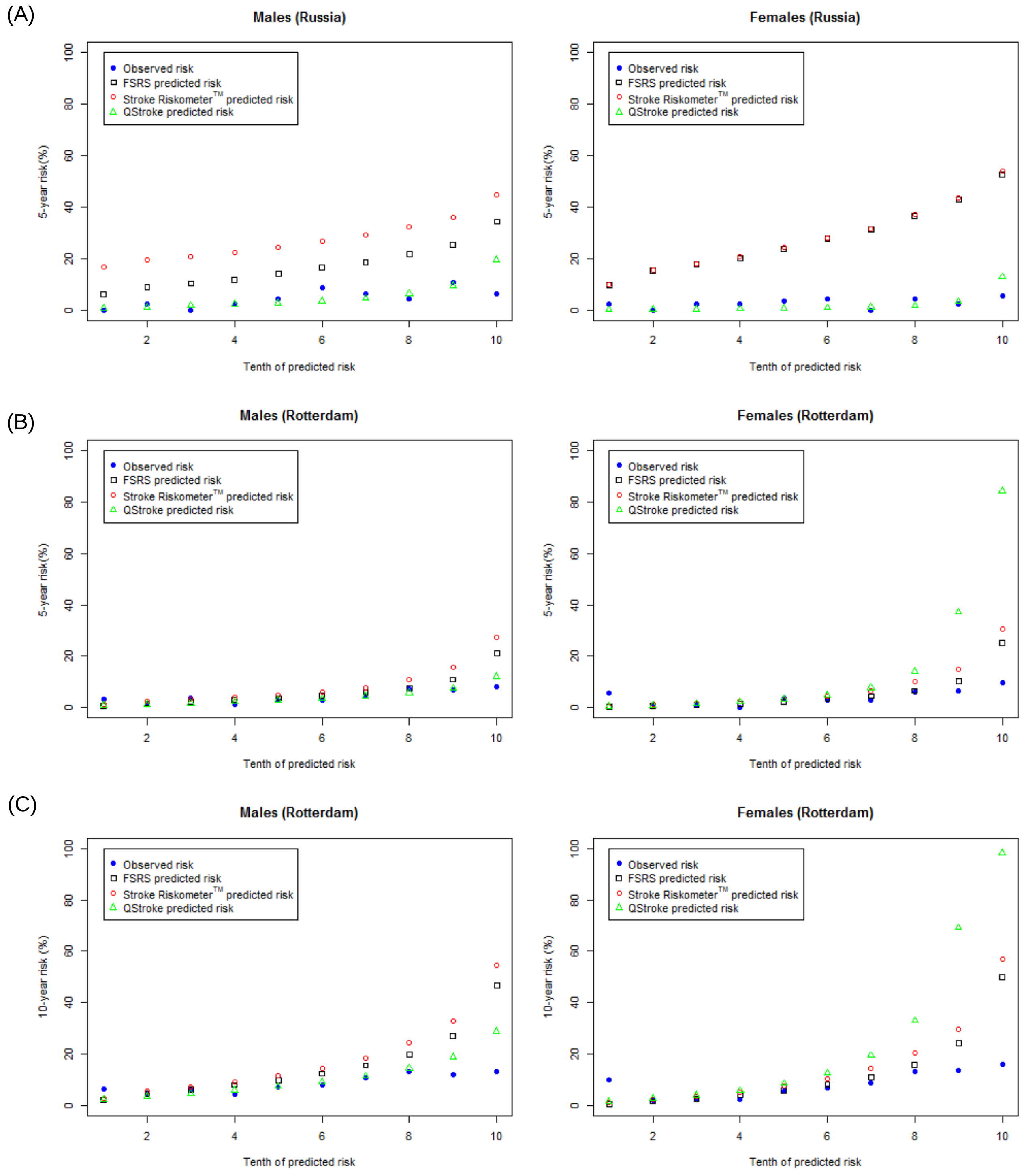

Supplement: Supplementary file 1 — Fig. S1. Bar plots of mean predicted risk scores for observed stroke and nonstroke events at (A) 5-years and (B) 10-years for FSRS (black), Stroke Riskometer™ (red) and QStroke (green) algorithms across the (1A) Rotterdam and (1B) combined [ARCOS, Russia and Rotterdam] data sets for males (left) and females (right). Fig. S2. Mean predicted risk (%) vs. observed stroke events in deciles of predicted risk for Framingham Stroke Risk Score (FSRS) (black), Stroke Riskometer™ (red) and QStroke (green) algorithms for males (left) and females (right) for (A) 5-year predicted risks for the Russian data set, (B) 5-year predicted risks for the Rotterdam data set and (C) 10-year predicted risks for the Rotterdam data set. [file ijs0010-0231-sd1.pdf]
